# “It’s not easy, but we need to support them so they don’t get tired on the way”: provider perspectives on delivering HIV services for people returning to HIV care in Cape Town, South Africa

**DOI:** 10.1186/s12913-026-14898-0

**Published:** 2026-06-09

**Authors:** Tamsin K. Phillips, Hannah Wolpe, Nasiphi Gwiji, Kate Clouse, Benjamin H. Chi, Suzanne Maman, Landon Myer, Lucia Knight

**Affiliations:** 1 Division of Epidemiology & Biostatistics, School of Public Health, University of Cape Town, Cape Town, South Africa; 2 Division of Social & Behavioral Sciences, School of Public Health, University of Cape Town, Cape Town, South Africa; 3 Vanderbilt University School of Nursing, Nashville, TN USA; 4 Vanderbilt Institute for Global Health, Nashville, TN USA; 5 Department of Obstetrics and Gynecology, School of Medicine, University of North Carolina at Chapel Hill, Chapel Hill, NC USA; 6 Department of Health Behavior, Gillings School of Global Public Health, University of North Carolina at Chapel Hill, Chapel Hill, NC USA; 7 School of Public Health, University of the Western Cape, Bellville, South Africa

**Keywords:** HIV, ART, Return to care, Providers, CFIR, South Africa

## Abstract

**Background:**

People living with HIV who interrupt treatment often face challenges when re-engaging in care and remain at risk for future interruptions. Understanding the provider perspective on the healthcare context in which re-engagement occurs is essential for designing effective and scalable support interventions. We aimed to characterize the implementation context for re-engaging in HIV care in Cape Town, South Africa, with a view to inform the design of future interventions in this setting.

**Methods:**

We conducted semi-structured in-depth interviews with nurses (*n* = 5), counsellors (*n* = 3) and administrative clerks (*n* = 3) at two public-sector health facilities. Data collection and thematic analysis were guided by the inner setting of the Consolidated Framework for Implementation Research (CFIR), including structural characteristics, organizational culture, relational dynamics, and communication.

**Results:**

We identified compounding and interconnected challenges within the CFIR inner setting. Limited staffing and fragmented clinic organization contributed to delays and frustration for both clients of HIV services and providers, reinforcing negative client-provider interactions. Despite valuing person-centred care, providers struggled to uphold these principles amid high workloads and systemic pressures relating to physical, information system, and human resource infrastructure limitations. Additionally, a cross-cutting theme was the challenge of inter-facility transfers, driven by geographic mobility and stigmatizing clinic interactions. Difficulties in linking client information across clinics intersected with the structural and relational barriers, adding another layer of complexity to providing supportive care for people returning to HIV services.

**Conclusions:**

Providers in this context were empathetic but layered inner setting barriers made it challenging to provide person-centred care. Addressing these challenges requires interventions that not only accommodate the changing life circumstances of people living with HIV but crucially facility- and system-level changes to support the providers and their working environments, which together will foster strong reciprocal client-provider relationships. Future interventions must prioritize strengthening staffing and workload capacity, physical infrastructure, and information systems alongside simplified, responsive service delivery models to ease the path back into care for people living with HIV and their providers.

**Supplementary Information:**

The online version contains supplementary material available at 10.1186/s12913-026-14898-0.

## Background

People living with HIV (PLH) on lifelong antiretroviral therapy (ART) often cycle out and back into care over time [1]. In South Africa, people re-initiating ART after an interruption represent a substantial proportion of those starting ART [2, 3]. However, even following re-initiation of ART, people returning remain vulnerable to repeat disengagement and suboptimal outcomes [4–6].

There is growing focus on interventions to re-engage PLH who have disengaged from care [7]; however, less attention has been given to sustaining engagement after return. Successful support interventions require an understanding of the context in which care is delivered to ensure approaches are acceptable, feasible, and scalable. In implementation science, this context—known as the “inner setting”—is shaped by frontline providers, who are critical to successful implementation [8]. The Consolidated Framework for Implementation Research (CFIR) provides a structure for examining implementation determinants within the inner setting, including structural characteristics, relational connections, communications, and culture [9].

Aligned with global calls for person-centred care (care which is respectful, responsive, and empowering, treating people living with HIV as whole individuals with complex needs and equal partners in coordinated care) [10–12], South Africa launched the Welcome Back Campaign in 2019 [13]. This national initiative used media, community engagement, and health service interventions to encourage people who had interrupted HIV treatment to return to care. Local implementing partners (non-governmental organisations [NGO]) who collaborate with the Department of Health (DoH) to support HIV service delivery through technical assistance and human resources) supported the campaign with activities varying across regions and clinics. In Cape Town NGO implementing partners supported provider training and sensitization, media outreach, and provided additional staff to support active tracing of people lost to follow-up and fast-tracked care for those returning. The goal was to facilitate easy re-engagement in care in a welcoming and nonjudgemental clinic environment.

Through in-depth interviews with nurses, HIV counsellors, and administrative clerks—frontline providers central to HIV service delivery—we aimed to characterize the service delivery context for PLH returning to care after treatment interruption in Cape Town, South Africa and generate insights to inform future intervention design and implementation in this setting.

## Methods

### Setting

This study took place in Gugulethu, a large township in Cape Town in the Western Cape province of South Africa where free HIV treatment has been available since 2004. Despite progress, the area continues to face high unemployment, poverty and HIV prevalence. According to the 2022 antenatal HIV sentinel survey, the Western Cape had the lowest HIV prevalence nationally (16%), but this varied widely by district (20% in Cape Town) [14]. There is no recent estimate from Gugulethu but a study from 2019 reported antenatal HIV prevalence was approximately 30% [15] and in 2023, program data showed nearly 9000 people were on ART at the two study clinics. At the time of the study all counselling staff were employed through NGO implementing partners.

We worked with the local department of health to select participating clinics. One large provincial clinic (clinic 1, ~ 7000 people on ART in 2022) and one smaller municipal clinic (clinic 2, ~ 2000 people on ART in 2022) were selected to provide variation in volume and clinic administration. At both study clinics, NGO implementing partner staff, including a Welcome Back nurse dedicated to seeing returning clients, counsellors and clerks, worked alongside department of health staff to provide routine HIV services and implement Welcome Back activities. Clients returning to care were fast tracked for clinical assessment with the Welcome Back nurse and re-initiated ART on the same day or, if needed, after blood test review [16]. Clients initially received a one-month ART supply, followed by a two-month supply at their next visit. An HIV viral load test was conducted after three months and those with viral suppression became eligible for differentiated service delivery models, such as adherence clubs, multi-month dispensing, and alternative ART pick-up points. Those not suppressed continued care in the clinic with enhanced counselling.

### Data collection

Following initial discussions with facility management, the study team attended a facility staff meeting to present the planned research and engaged with providers to understand clinic workflows. Healthcare providers were invited to participate in semi-structured in-depth interviews exploring their perceptions of HIV service delivery for people returning to care. Providers were eligible to participate if their role involved any aspect of care for clients returning to HIV services. We used purposive sampling to identify NGO and department of health staff implementing the Welcome Back campaign and routine HIV care. The researchers had no prior relationship with potential staff. Facility managers helped to identify appropriate staff following which the interviewer spoke to providers one-on-one at the facility to provide more information on the study and assess willingness to participate. Those interested completed written informed consent. All invited providers agreed to participate. Interviews lasting 30 to 60 min were conducted during working hours at a time convenient for participants. All interviews were audio recorded and took place in a private room in the clinic. Interviews were conducted by a female researcher (NG, MPH student in Social and Behavioural Sciences employed as qualitative research assistant) fluent in isiXhosa and English, with training and experience in qualitative methods. The interviewer wrote notes after each interview. A research assistant fluent in isiXhosa and English transcribed the audio recordings into English for analysis. All identifiers were removed in transcription and a second research assistant verified transcript accuracy before coding.

The interview guide (Supplemental material 1) was informed by the CFIR’s inner setting domain [9], which we defined as the clinic environment where HIV care was delivered. We focused on the persistent characteristics that exist regardless of implementation of any specific intervention as these would be most relevant in broad considerations for new interventions (Table 1). Questions focused on experiences and perspectives supporting clients who return to the clinic who have interrupted HIV care, including available services and service structure, resources and infrastructure, and communication networks within and between clinics. After initial interviews, the guide was revised to simplify language and remove elements not relevant to non-management staff. Transcripts were not returned to participants for comment.

### Data analysis

Interviews were thematically analysed following Braun and Clarke’s six-step framework [17]. While we did not frame our sample size in terms of saturation, recurring themes across the 11 provider interviews indicated that the data were sufficient to address our research aim. The primary analyst (HW) first familiarized themselves with the data by repeatedly reading transcripts and the interview notes (step 1) before developing and applying initial codes (step 2), which were then grouped into themes (step 3). Steps 1–3 were completed without reference to the CFIR constructs. A second investigator (TP) thoroughly reviewed transcripts, analyses, and reviewed thematic interpretations through iterative debriefing with the interviewer and primary analyst (step 4). In step 4, we began organising the results according the CFIR inner setting. Using *NVivo* software, the analysis followed an iterative process, combining inductive and deductive coding based on the study’s research questions and interview guide. Emerging themes were further reviewed and refined (step 5) by co-authors with varying degrees of familiarity with the context and in this step, we completed organization using CFIR’s inner setting constructs [9]. HW and TP produced the initial report with data extracts and analysis which was reviewed and refined by all co-authors (step 6). It was in step 5 that inter-facility transfers was pulled out as a cross-cutting theme that did not fit directly within the inner setting constructs. With the exception of verbatim provider quotes which refer to *patients*, we use the term *client* throughout for clarity and consistency, while drawing on the CFIR construct of “recipient-centredness” to frame care experiences of HIV service clients.

Selected illustrative quotes are provided in the text. Extended quotes retaining additional context and additional quotes are presented in Supplemental Material 2 to enhance transferability and credibility, allowing readers to interpret the text independently. This study was reported in accordance with the Consolidated Criteria for Reporting Qualitative Research (COREQ) checklist (Supplemental Material 3) [18].

**Table 1:** Description of relevant constructs in the inner setting domain of the Consolidated Framework for Implementation Research (CFIR). Descriptions are taken directly from the published updated CFIR (Damschroder et al. Implementation Science (2022) 17:75)

Inner setting	The setting in which the innovation is implemented
Structural characteristics	Infrastructure components support functional performance of the Inner Setting
Work infrastructure	Organization of tasks and responsibilities within and between individuals and teams, and general staffing levels.
Physical infrastructure	Layout and configuration of space and other tangible material features
Information Technology (IT) infrastructure	Technological systems for tele-communication, electronic documentation, and data storage, management, reporting, and analysis support
Organizational culture	Shared values and basic assumptions that explain why organizations do what they do and focus on what they focus on
Human equality	The recognition of all individuals’ inherent equal worth and value
Recipient-centeredness	The shared values, beliefs, and norms guiding care, support, and responsiveness to recipients’ needs
Relational connections	Formal and informal relationships, networks, and teams within and across Inner Setting boundaries
Communication	Formal and informal information sharing practices within and across Inner Setting boundaries

### Ethics

This study was conducted in accordance with the principles of the Declaration of Helsinki. The protocol was approved by the University of Cape Town Human Research Ethics Committee (ref 305/2021), the Western Cape Department of Health and Wellness, and the City of Cape Town Health Department. Written informed consent was obtained from all participants before data collection.

## Results

We enrolled five nurses (two NGO [Welcome Back nurses] and three DoH staff [including two nurse managers]), three HIV counsellors (all NGO), and three administrative clerks (two DoH, one NGO) between March 2022 and April 2023. Most participants were female, age ranged from 27 to 58 years, and participants had 2–20 years of experience working in HIV services (Table 2).

This analysis examines the factors that enable or hinder the implementation of supportive HIV care for clients returning to care. We identified a cascade of interconnected challenges within the CFIR inner setting, spanning structural characteristics, organizational culture, relational dynamics, and communication. Additionally, a distinct but cross-cutting theme emerged around inter-facility transfers.

**Table 2:** Characteristics of providers interviewed

	Total	Nurses	Counsellors	Administrative clerks
Number of participants	11	5	3	3
Gender, n				
Female	8	3	3	2
Male	3	2	0	1
Age, median (range)	45 (27–58)	50 (42–54)	47 (32–58)	44 (27–45)
Years of experience working in HIV, median (range)	13 (2–20)	10 (3–15)	17 (13–20)	7 (2–16)
Study clinic, n				
Clinic 1 - Large	7	3	2	2
Clinic 2 - Small	4	2	1	1

### Structural characteristics

Participants described service delivery conditions as resource-constrained, limiting their ability to support clients returning to care. Key structural challenges included staff shortages, space limitations, and technological constraints. Staff training was seen as both essential and a contributor to staff absences.

#### Work infrastructure

Insufficient staffing and unfilled posts were major barriers to service implementation. Absenteeism, particularly in winter due to illness, further compounded these challenges. Staff shortages led to delays in client care and increased pressure on available providers. The frustration clients experienced when not attended to promptly was seen as a barrier to their continued engagement in care.

… absenteeism of staff. Let me give an example: We’ve got booked patients that are 110 for today. Then the turnout of the staff… I’ve got only 2 sisters. That will mean, if I’ve got an extra load, it was… A sister can only see up to 35. How to do it now… The 70 out of the 110 that we have got… There’s an extra… extra patients that I don’t know, who will see them. They must stretch themselves. And that thing doesn’t make them very nice…They’re not happy at all at that time. And if they overstretch themselves then, be sure tomorrow they’re not coming back. (Nurse Manager, clinic 1)

Due to staff shortages, providers often took on tasks beyond their usual roles, such as counsellors translating conversations between doctors and clients or nurses providing counselling. Counsellors in particular expressed frustration over the intense time pressures they faced.

Sometimes the doctors or the nurses will say, “no, you’re spending too much time with the patients” you see… And now for us a counsellor, it’s a need; we see that for us, it helps the patient but for you [the doctor] it’s all about time! (Counsellor, clinic 1)

While essential, staff training reduced the number of providers available to see clients. Training was paused during the COVID-19 pandemic, and efforts to catch up afterward led to significant but temporary staff shortages.

…And there’s also been a holdup on trainings for the staff… due to COVID related issues. Now we’ve started again. That means now we don’t always have full capacity of staff… We are here but now we must let half of the people go to trainings… because we need training. (Nurse Manager, clinic 1)

Conversely, some providers emphasized the need for additional training beyond HIV-specific issues, including noncommunicable diseases, and strategies to foster more positive provider attitudes. One administrative clerk called for “*in-service training of staff making sure that they are on par with what are the needs of the clients and also working on the attitude of the staff”.*

Although fast-tracked services routed returning clients to a dedicated Welcome Back nurse, their implementation was inconsistent due to the challenges described above. Absenteeism and staffing changes, including temporary agency staff, disrupted continuity of care and the consistent delivery of fast-tracked services. Providers also noted that long waiting times—exacerbated by staffing shortages and pharmacy queues—led to frustration, which could contribute to disengagement from care.

[The Welcome Back nurse] is not always available, they [patients] will be seen by someone else and that’s another disappointment, they will see that they are not welcomed. I try to assist when they are not around but it’s not going to work because I am working through an agent. I might be here this month and not be here again next month. (Nurse, clinic 2)

The organization of tasks and clinic workflow was reported to be further disrupted by returning clients presenting as newly diagnosed or when returning clients were very ill and had other health needs. Providers reported that clients presenting as new would not be fast-tracked as intended and may still face delays if later identified as returning. Very ill clients could not be fast-tracked as they needed additional clinical care. While this points to individual-level client factors that delay or complicate return to care including anticipated stigma and delays associated with having dropped out of care, this also highlights that some workplace challenges are driven by the individual needs, perceptions and circumstances of the clients.

They [the clients] don’t know the system, they don’t know that they will be fast tracked, some lie and don’t say they have interrupted treatment. They first go and test and bring a new letter. You only realize this patient was already on treatment when you capture it. Those are the challenges because they are scared of how they will be treated after an interruption… Others would say I was scared because you shout, so I decided to be a new patient. (Administrative Clerk, clinic 1)

These findings highlight how limited staffing and fragmented clinic organization were perceived to contribute to delays, pressures and a cycle of frustration for both clients and providers.

#### Physical & Information Technology (IT) infrastructure

Concerns about physical and IT infrastructure were most prominent in the larger of the two clinics. Providers cited a lack of computers and unreliable internet access as major sources of frustration, leading to delays in care. To avoid waiting for a computer, some used personal mobile phones to access blood results. Limited physical space also posed challenges, with nurses having to relocate their workstations when the temporary doctor was on site, and counsellors having to share rooms due to lack of dedicated counselling spaces.

A lack of computers… we have to retrieve the results from our phones… because we’ve got too few PC’s… we end up losing our [personal phone] data instead because… to save time. Because those results, we need in order to see the direction [for the patient]. (Nurse, clinic 1)

Nurses emphasized the importance of being able to easily access client folders and treatment histories when restarting ART. At both clinics, poor coordination of filing and difficulty locating client folders led to confusion and delays in care.

Duplicated folders is the main issue [when the primary folder is not found]… And each and every one [staff] is pushing around this folder of a duplicate… Not interested to see it because there are lots of things that aren’t there. Information that is missing. (Nurse Manager, clinic 1)

To minimize disengagement, some participants suggested implementing a system to track folder locations within clinics to reduce delays and ensure access to client information. Separate filing and information systems—both between clinics with different administrations and within different sections of the same clinic—were seen as problematic. The Western Cape’s electronic medical record system, Single Patient Viewer (SPV), does connect public sector clinics across the province, but use was limited due to poor IT infrastructure. An administrative clerk highlighted the need for a nationally integrated information system.

Number one [problem] for me is the staff. Secondly, I think it’s the programs, the system [information systems] that we are using don’t link into each other. For example, if you took treatment at [another clinic] I can’t see, I don’t have a link. I can’t see that you were talking about treatment there. (Administrative clerk, clinic 1)

Insufficient computers and unreliable internet access prevented effective use of the SPV, which could have streamlined care for returning clients. Limited staff further compounded inefficiencies in folder systems and ability to locate client records. Workplace, physical, and IT infrastructure challenges thus intersected, creating delays and frustration across multiple aspects of care for clients returning to care.

### Organizational culture

The construct of culture in the CFIR attempts to capture the “shared values and basic assumptions that explain why organizations do what they do and focus on what they focus on” [19]. While aspects are interwoven throughout the other themes discussed, two key cultural dimensions emerged: human equality (i.e., the recognition of all individuals’ inherent equal worth and value) and recipient-centeredness (i.e., the shared values, beliefs, and norms guiding care, support, and responsiveness to the needs of clients receiving care).

Provider attitudes toward clients returning to care reflected a tension between empathy and frustration, particularly for those who repeatedly cycled in and out of care. Navigating this tension within the structural constraints mentioned above, shaped provider behaviours and attitudes towards clients.

I applaud those who return because this journey is tough. I can imagine taking treatment very often. They need to be loved, they need to be cared for, they need to be told that we care for you…It’s not easy, but we need to support them so they don’t get tired on the way. (Administrative clerk, clinic 2)

Why could a person, every time, become pregnant, stop treatment, restart… like for the third time, you know? But then… then again, I think there are a lot of things going through people. (Nurse, clinic 1)

Despite this tension, all providers emphasized the importance of creating an environment where clients feel heard and supported. They highlighted the need to foster supportive relationships at every point of interaction with the health service. However, both actual and anticipated poor care experiences—including rude providers and long wait times for those without appointments—were seen as common reasons for repeat disengagement and failure to return to care.

We still get one or two who are still shouting at the clients and I think none of us would want to be shouted at whether [or not] I have done something wrong. I want somebody to speak to me in a nice way… So, we need to have a listening ear to clients for the problems or challenges that they might have encountered and try to engage with them so that we shouldn’t have the same problem and go back to defaulting their treatment… (Nurse, clinic 2).

Taken together, providers expressed frustrations that influenced their responses to clients, despite expressing a strong belief in the equal worth of all individuals and a commitment to person-centred, trusting relationships with clients returning to care.

### Relational connections and communication

The strength of relational connections varied and was closely linked to the quality of communication among providers and between providers and clients. All providers emphasized the importance of collaboration in delivering supportive care and ensuring continued engagement. At both clinics, providers highlighted the value of multidisciplinary team (MDT) meetings. One nurse described these meetings as platforms “*for people to raise their concerns or challenges and things can be resolved before they go further*.” A team-based approach was seen as essential for improving communication, strengthening support across staff levels, and enhancing client care.

We say we are a team of five in their treatment journey… It’s the client, treatment buddy, doctor, counsellor and a nurse. So, they need to know that in order for their lives to be better, we should be a team of five. They also need to be involved in order for their lives to be good. (Counsellor, clinic 2)

There was a general sense that district and sub-district leadership were aware of the challenges in providing care for returning clients. However, two nurses spoke of not receiving adequate support to implement change.

They [sub-district leadership] always emphasize, they always report that: You’ve got a high number of defaulters. What is happening? What interventions must we apply? Because it’s not like they’re coming up with plans… like, what can we do now? (Nurse manager, clinic 1)

In both clinics, non-governmental implementing partners employed counsellors, administrative clerks, and nurses to work in the clinic alongside the DoH staff to support service provision. One counsellor noted a disconnect between the clinic activities and those of the implementing partner organizations. They expressed a sense that decision-making primarily took place among clinical providers and clinic management, despite the implementing partner counsellors carrying a significant load of client support.

… they have staff meetings that we are not invited to… it’s almost like when they come to us as counsellors, it’s always complaints more than acknowledgement… (Counsellor, clinic 1).

Providers at one clinic emphasized the need for inclusive discussions among all facility staff, specifically highlighting challenges with restricted facility access for clients returning to the clinic without appointments. Clients traced telephonically were instructed to call a clerk or counsellor to be escorted inside, but clients returning to care on their own risked being turned away if communication between security staff and providers was lacking. While access restrictions were implemented for the COVID-19 pandemic, many remained in place.

But the main issue, because of the COVID-related issues, is the access at the gate… because another patient would say, “I’m sick, I came here 2 days ago, then I was told I don’t have an appointment, so I couldn’t access [the clinic].” …we don’t see people by dates, but now they will turn you [away] at the gate. (Nurse manager, clinic 1)

Overall, the strength of formal and informal communication among all staff interacting with returning clients—from arrival at the clinic, through reception, counselling, clinical care, medication dispensing and folder storage—was seen to influence providers and client experiences with return to care.

### Inter-facility transfers

Inter-facility transfer challenges emerged across inner setting constructs but did not fit squarely within this domain. Human mobility for economic and personal reasons intersects multiple CFIR domains, with knock-on health service responses embedded in the inner setting [9]. Inner setting characteristics are critical for adaptable systems that sustain HIV care, including streamlined transfers during mobility. Thus, inter-facility transfer is presented as a cross-cutting theme that has distinct implications for continuity of care for returning clients.

Clinic transfers created challenges in accessing clients’ treatment history, which providers viewed as essential for delivering appropriate care moving forward. Clients being required to present a transfer letter posed a barrier to continuity of care. Linked electronic health information systems in the Western Cape helped bridge this gap but did not eliminate provider need for a transfer letter. Movement to and from provinces beyond the Western Cape added further complexity, with access to treatment history often left to the discretion of the receiving provider. Providers could call a clinic to request a letter via email, but this hinged on whether a staff member was willing to assist. Some providers recalled instances where clients’ relatives had sought to obtain transfer letters but were turned away, or where clients lacked the funds to travel back to their original clinic to request a letter themselves.

As I said our patients are very mobile, they will move from one area to another. They relocate and then leave and not come back to request a transfer letter… because when you get to the other side, wherever you have moved to, if you don’t produce a transfer letter it’s difficult for them to say what treatment are you on and whether to continue or to restart you on treatment because they don’t know exactly what treatment you are using. (Nurse, clinic 2)

Clinic transfers within and outside the Western Cape were seen to driven by geographic mobility, changing circumstances, and stigma. Providers noted that perceived or experienced stigma often led clients to switch clinics to avoid being recognized. The December holiday period, when many travel across South Africa for family gatherings, was associated with high dropout rates and low return to care. When in a new area, clinic inaccessibility and stigma, for those who had not disclosed their status, was seen to further hinder care continuity for mobile clients.

So, the movement is also a problem… So, it depends on the clerk assisting the patient because someone would say it’s not my problem that they don’t have a letter. (Administrative Clerk, Clinic 1)

Providers also raised concerns about clinic catchment areas and the requirement for clients to provide proof of residence. Some clients, seeking care at a preferred clinic for personal reasons, provided false addresses to meet eligibility criteria to attend the clinic of their choice. Providing incorrect addresses was reported as a considerable challenge for the clinic staff attempting to recall people with missed visits.

They come here and give us local addresses whereas they don’t live here in the area because they like this clinic. Maybe they live in [another area] and we can’t go there. (Administrative clerk, clinic 2)

Or maybe they prefer that “I used to take my treatment from a different facility and now I want to take it from this facility.” Right, they get there and they [the clerk] can see on the system… Maybe I want to get my treatment now the system is saying “go back and get a transfer!” How will they come back? (Counsellor, clinic 1)

This lack of service adaptability for mobile clients heightened frustrations for both clients and providers. Difficulties linking client information across clinics intersected with underlying structural and relational barriers within the inner setting, adding another layer of complexity to providing supportive care for people returning to HIV services.

## Discussion

In this setting, where providers had been sensitized to a person-centred approach and efforts had been made to make HIV services more welcoming and accessible, several barriers continued to hinder the implementation of supportive care for people returning to HIV treatment. While cultural and relational barriers are persistent and frequently cited as determinants of poor treatment continuity, our results highlight that successful intervention in these areas is likely to be contingent on addressing broader structural constraints. Despite strong commitment and understanding, layered and compounding structural and relational challenges shaped provider ability to provide person-centred care.

Inter-facility transfer—driven by mobility, changing life circumstances and stigma—emerged as an important theme cutting across inner setting constructs and adding a valuable provider perspective to literature. Previous research in South Africa and other LMICs has shown that geographic mobility is a key contributor to disengagement from HIV care [20, 21]. People may switch clinics to avoid a facility where they previously experienced provider stigma or to protect their status from being disclosed in their community [20, 22, 23]. Mobility can also prevent engagement in care due to access challenges and anticipated stigma, when people fear negative attitudes from providers when trying to access unfamiliar clinics [20, 24]. Our results underscore the need for more adaptable, client-responsive health systems, including greater interconnectivity between clinics across local and provincial boundaries. Rather than treating mobility as a disruption, the health system must come to expect that people will experience changes in employment, housing, and relationships over time, and build flexibility into care pathways to enable continued access to HIV services through these transitions.

Linked to this was the persistent challenge of accessing clinical history for PLH returning to care after an interruption. Although South African and local ART guidelines clearly state that no one may be denied ART in the absence of a transfer letter [16, 25], providers reported that this still occurs in practice, resulting in frustration for both clients and staff. Enhancing clinic connectivity through facility-linked electronic medical records could reduce the barriers imposed by transfer letters and enable smooth re-initiation of care. Our data and findings from other studies suggest that some ART-experienced clients present as “new” clients, possibly to avoid the stigma of having interrupted treatment or to escape anticipated delays and requirements associated with re-engagement [3, 26]. The absence of a transfer letter may become less critical at the point of re-initiation in the context of dolutegravir-based regimens which are recommended as first- and second-line ART in South Africa [16, 27]. Participatory research is needed to define the minimum set of treatment history information required to safely restart ART and the simplest avenues to access this information, while ensuring the health system supports, rather than punishes, mobility and flexibility for PLH.

Negative client-provider interactions are a well-established factor influencing engagement in HIV care [21]. PLH consistently express a preference for friendly, empathetic providers and may even tolerate longer wait times in exchange for a more supportive clinic environment [28]. Compassionate, respectful, and person-centred care has been identified as a defining characteristic of clinics with higher retention rates in South Africa [29], echoing the central principles of the 2024 World Health Organization policy brief on re-engagement in HIV care [30]. Our findings align with prior qualitative research showing that providers recognize the importance of their attitudes in fostering ongoing engagement [31]. However, awareness alone is not sufficient. An evaluation of a “Welcome Service” intervention in Cape Town found that while providers understood the value of person-centred care, this did not always translate into less judgmental behaviour [32]. Similarly, some providers in our study reported being blamed for colleagues’ less compassionate interactions, underscoring how individual relationships are shaped by the broader culture of care within facilities. A person-centred intervention for men living with HIV in Malawi addressed this by included an apology for past negative experiences as a core intervention element in attempt to rebuild client-provider trust [33]. Given the persistence of stigma and the growing population of long-term ART-experienced PLH, strategies that are sensitive to past negative experiences and that ease structural challenges will be particularly valuable.

In addition to the above, our findings crucially demonstrate that interventions to improve client-provider relationships alone are unlikely to succeed without upstream investment in work, physical and IT infrastructure. While these barriers are deeply embedded and difficult to modify [34, 35], this investment will be essential to sustainably counteract providers’ understandable responses to existing working environments and the broader challenges of the systems and communities’ they work in. The CFIR framework highlights that the shared values and norms around caring for providers is a key element of culture in the inner setting [9]. While providers in our study did not explicitly call for this, our findings suggest that some degree of provider-centeredness is essential to enable the delivery of person-centred care. Beyond the infrastructure issues we have documented, challenges related to job security, remuneration, and safety are well known in the Western Cape and many other LMIC settings [36, 37]. Weak relationships and communication between all staff cadres, including those employed by implementing partners, also hampers connection and workplace trust [38]. Prior research shows that when providers’ basic needs are met—and when morale, teamwork, and intrinsic motivation are high—retention in HIV care improves [29, 38, 39]. However, most interventions to improve client-provider relationships in LMICs have focused narrowly on changing provider behaviour, for instance, through sensitization, communication training, or support for individualized counselling [40]. Interventions focused solely on provider training or sensitisation are unlikely to be effective in the absence of changes to workload, information systems, and service organisation. Structural intervention and attending to professional quality of life may be a critical, and underutilized, pathway to improving both provider well-being and client care outcomes.

Our findings highlight key implications for the design and implementation of support interventions for clients returning to HIV care. Health systems should avoid approaches that focus narrowly on changing provider attitudes without simultaneously addressing the structural constraints that shape their daily practice. Providers cannot be expected to sustainably deliver person-centred care unless they are equipped with the necessary resources, infrastructure, and support. At the system level, priorities should include strengthening infrastructure to enable continuity of care within and across facilities, and transparently managing workload constraints through clear delineation of roles and responsibilities for all staff cadres, including those from the DoH and implementing partners. These system priorities intersect with facility-level needs such as improving access to client information across sites, clarifying provider roles, and fostering transparent communication within and between provider teams.

These findings should be interpreted considering several limitations. This research represents the context in two public sector clinics in peri urban Cape Town, South Africa. The work was intended to generate insights to inform future intervention design and implementation and does not intend to reflect the implementation context in all South African clinics. However, themes such as resource availability and transfer challenges are likely to be more broadly relevant. This study presents perspective of providers working in the clinics and while we have drawn some insights into implementing partner roles and challenges, this was not a core focus of this work. Further research with a wider group of stakeholders, including managers, implementing partners, funders and policy makers, will be essential to inform structural changes needed to support providers to deliver person-centred care. Finally, this work reflects the context prior to the 2025 withdrawal of US donor funding in South Africa. Many of the principles of the Welcome Back campaign have been adopted by health services and they are encompassed in the current Closing the Gap campaign, a strategic initiative focused on reaching the UNAIDS 95-95-95 targets [41]. Shifts in the South African HIV care landscape, including the removal or downscaling of many implementing partners who supported client tracing, counselling, and data monitoring has likely exacerbated many of the challenges identified in this study and imposed additional barriers [42].

## Conclusion

It is important to recognize that disengagement and re-engagement are often part of the lifelong journey of HIV treatment, rather than deviations from it. While reducing periods of disengagement remains critical, our findings highlight an equally urgent need to ensure that re-entry into care is met with supportive, compassionate, and efficient services. However, barriers within the inner setting including workplace, physical and IT infrastructural limitations, poor-quality formal and informal communication, and challenging organisational culture, limit the responsiveness of health services and place substantial pressure on providers who may understandably respond negatively. Future interventions must prioritize simplified, responsive service delivery models and structural strategies that not only ease the path back into care for people living with HIV, but crucially support the providers tasked with walking that path alongside them.

## Supplementary Information

Below is the link to the electronic supplementary material.

Supplementary Material 1

Supplementary Material 2

Supplementary Material 3

## Data Availability

The qualitative dataset used and/or analysed during the current study is not publicly available in a repository. All relevant data are described within the manuscript itself.

## References

[1] Ehrenkranz P, Rosen S, Boulle A, Eaton JW, Ford N, Fox MP, et al. The revolving door of HIV care: Revising the service delivery cascade to achieve the UNAIDS 95-95-95 goals. PLoS Med. 2021;18:e1003651. 10.1371/journal.pmed.1003651. 34029346 10.1371/journal.pmed.1003651 PMC8186775

[2] Euvrard J, Timmerman V, Keene CM, Phelanyane F, Heekes A, Rice BD, et al. The cyclical cascade of HIV care: Temporal care engagement trends within a population-wide cohort. PLoS Med. 2024;21:e1004407. 10.1371/journal.pmed.1004407. 38728361 10.1371/journal.pmed.1004407 PMC11125544

[3] Benade M, Maskew M, Ntjikelane V, Scott N, Ngcobo N, Nichols B, et al. Prior antiretroviral therapy exposure among clients presenting for HIV treatment initiation in South Africa: an exploratory mixed-methods study using multiple indicators of exposure. BMC Infect Dis. 2025;25:947. 10.1186/s12879-025-11340-4. 40713520 10.1186/s12879-025-11340-4 PMC12296601

[4] Nakiwogga-Muwanga A, Musaazi J, Katabira E, Worodria W, Talisuna SA, Colebunders R. Patients who return to care after tracking remain at high risk of attrition: experience from a large HIV clinic, Uganda. Int J STD AIDS. 2015;26:42–7. 10.1177/0956462414529098. 24648320 10.1177/0956462414529098

[5] Rana AI, Liu T, Gillani FS, Reece R, Kojic EM, Zlotnick C, et al. Multiple gaps in care common among newly diagnosed HIV patients. AIDS Care. 2015;27:679–87. 10.1080/09540121.2015.1005002. 25634492 10.1080/09540121.2015.1005002 PMC4366312

[6] Moolla H, Davies MA, Davies C, Euvrard J, Prozesky HW, Fox MP, et al. The effect of care interruptions on mortality in adults resuming antiretroviral therapy. AIDS. 2024;38:1198–205. 10.1097/QAD.0000000000003859. 38814712 10.1097/QAD.0000000000003859 PMC11141523

[7] Mirzazadeh A, Eshun-Wilson I, Thompson RR, Bonyani A, Kahn JG, Baral SD, et al. Interventions to reengage people living with HIV who are lost to follow-up from HIV treatment programs: A systematic review and meta-analysis. PLoS Med. 2022;19:e1003940. 10.1371/journal.pmed.1003940. 35290369 10.1371/journal.pmed.1003940 PMC8923443

[8] Damschroder LJ, Aron DC, Keith RE, Kirsh SR, Alexander JA, Lowery JC. Fostering implementation of health services research findings into practice: A consolidated framework for advancing implementation science. Implement Sci. 2009;4:1–15. 10.1186/1748-5908-4-50. 19664226 10.1186/1748-5908-4-50 PMC2736161

[9] Damschroder LJ, Reardon CM, Widerquist MAO, Lowery J. The updated Consolidated Framework for Implementation Research based on user feedback. Implement Sci. 2022;17:75. 10.1186/s13012-022-01245-0. 36309746 10.1186/s13012-022-01245-0 PMC9617234

[10] World Health Organization. Consolidated guidelines on person-centred HIV patient monitoring and case surveillance. Geneva, Switzerland: WHO; 2017.

[11] Grimsrud A, Wilkinson L, Eshun-Wilson I, Holmes C, Sikazwe I, Katz IT. Understanding engagement in HIV programmes: how health services can adapt to ensure no one is left behind. Curr HIV/AIDS Rep 2020;17:458–66. 10.1007/s11904-020-00522-1. 10.1007/s11904-020-00522-1 PMC7497373 32844274

[12] International AIDS Society. Approaches to implementation of person-centred care for people living with HIV in low-resource settings [Internet]. 2024 [cited 2026 Apr 8]. https://plus.iasociety.org/webcasts/approaches-implementation-person-centred-care-people-living-hiv-low-resource-settings.

[13] South African National Department of Health. The South African National Welcome Back Campaign Strategy. Pretoria: National Department of Health; 2021.

[14] Kufa T, Shangase N, Lombard C, Manda S, Puren A. The 2022 Antenatal HIV Sentinel Survey: key findings. Johannesburg: National Institute for Communicable Diseases; 2024.

[15] Kalk E, Heekes A, Slogrove AL, Phelanyane F, Davies M-A, Myer L, et al. Cohort profile: the Western Cape Pregnancy Exposure Registry (WCPER). BMJ Open. 2022;12:e060205. 10.1136/bmjopen-2021-060205. 35768089 10.1136/bmjopen-2021-060205 PMC9244673

[16] South African National Department of Health. 2023 ART clinical guidelines for the management of HIV in adults, pregnancy and breastfeeding, adolescents, children, infants and neonates. Pretoria: National Department of Health; 2023.

[17] Braun V, Clarke V. Using thematic analysis in psychology. Qual Res Psychol. 2006;3:77–101. 10.1191/1478088706qp063oa.

[18] Tong A, Sainsbury P, Craig J. Consolidated criteria for reporting qualitative research (COREQ): a 32-item checklist for interviews and focus groups. Int J Qual Health Care. 2007;19:349–57. 10.1093/INTQHC/MZM042. 17872937 10.1093/intqhc/mzm042

[19] Schneider B, González-Romá V, Ostroff C, West MA. Organizational climate and culture: Reflections on the history of the constructs in the Journal of Applied Psychology. J Appl Psychol. 2017;102:468–82. 10.1037/apl0000090. 28125256 10.1037/apl0000090

[20] Bisnauth MA, Davies N, Monareng S, Buthelezi F, Struthers H, McIntyre J, et al. Why do patients interrupt and return to antiretroviral therapy? Retention in HIV care from the patient’s perspective in Johannesburg, South Africa. PLoS ONE. 2021;16:1–15. 10.1371/journal.pone.0256540. 10.1371/journal.pone.0256540 PMC8412245 34473742

[21] Burke RM, Rickman HM, Pinto C, Ehrenkranz P, Choko A, Ford N. Reasons for disengagement from antiretroviral care in the era of Treat All in low-or middle-income countries: a systematic review. J Int AIDS Soc. 2024;27:26230. 10.1002/jia2.26230/full. 10.1002/jia2.26230 PMC10945039 38494657

[22] Camlin CS, Charlebois ED. Mobility and its effects on HIV acquisition and treatment engagement: recent theoretical and empirical advances. Curr HIV/AIDS Rep. 2019;16:314. 10.1007/S11904-019-00457-2. 31256348 10.1007/s11904-019-00457-2 PMC6663312

[23] Taylor BS, Reyes E, Levine EA, Khan SZ, Garduño LS, Donastorg Y, et al. Patterns of geographic mobility predict barriers to engagement in HIV care and antiretroviral treatment adherence. AIDS Patient Care STDS. 2014;28:284–95. 10.1089/apc.2014.0028. 24839872 10.1089/apc.2014.0028 PMC4046197

[24] Treatment Action Campaign. “I am very scared to go there without a transfer letter; I’m scared they will turn me away” [Internet]. 2025 [cited 2026 Jun 2]. https://www.tac.org.za/news/i-am-very-scared-to-go-there-without-a-transfer-letter-im-scared-they-will-turn-me-away/ https://www.tac.org.za/news/i-am-very-scared-to-go-there-without-a-transfer-letter-im-scared-they-will-turn-me-away/.

[25] Western Cape Government. Circular no. 78 of 2015: migration SOP. Cape Town: Western Cape Government; 2015.

[26] Sithole N, Gunda R, Koole O, Krows M, Schaafsma T, Moshabela M, et al. Undisclosed Antiretroviral Therapy Use at Primary Health Care Clinics in Rural KwaZulu Natal South Africa: A DO-ART Trial Sub-study. AIDS Behav. 2021;25:3695–703. 10.1007/s10461-021-03319-4. 34097208 10.1007/s10461-021-03319-4 PMC8560667

[27] Moorhouse MA, Carmona S, Davies N, Dlamini S, van Vuuren C, Manzini T, et al. Southern African HIV Clinicians Society Guidance on the use of dolutegravir in first-line antiretroviral therapy. South Afr J HIV Med. 2018;19:a917. 10.4102/sajhivmed.v19i1.917. 10.4102/sajhivmed.v19i1.917 PMC6244149 30473877

[28] Eshun-Wilson I, Kim HY, Schwartz S, Conte M, Glidden DV, Geng EH. Exploring Relative Preferences for HIV Service Features Using Discrete Choice Experiments: a Synthetic Review. Curr HIV/AIDS Rep. 2020;17:467–77. 10.1007/s11904-020-00520-3. 32860150 10.1007/s11904-020-00520-3 PMC7497362

[29] Ober AJ, Skinner DH, Bogart LM, Busakwe L, Davids W, Mahomed H, et al. Using positive deviance to enhance HIV care retention in South Africa: development of a compassion-focused program to improve the staff and patient experience. BMC Global Public Health. 2025;3:8. 10.1186/s44263-025-00123-3. 39910645 10.1186/s44263-025-00123-3 PMC11800582

[30] World Health Organization. Supporting re-engagement in HIV treatment services: policy brief. Geneva: World Health Organization; 2024.

[31] Nhemachena T, Späth C, Arendse KD, Lebelo K, Zokufa N, Cassidy T, et al. Between empathy and anger: healthcare workers’ perspectives on patient disengagement from antiretroviral treatment in Khayelitsha, South Africa - a qualitative study. BMC Prim Care. 2023;24:34. 10.1186/s12875-022-01957-8. 36698083 10.1186/s12875-022-01957-8 PMC9878968

[32] Arendse KD, Walker C, Pfaff C, Lebelo K, Cassidy T, Isaakidis P, et al. Supporting re-engagement with HIV services after treatment interruption in South Africa: a mixed method program evaluation of MSF’s Welcome Service. Sci Rep. 2024;14(1):7317. 10.1038/s41598-024-57774-9. 10.1038/s41598-024-57774-9 PMC10973441 38538754

[33] Hubbard J, Mphande M, Robson I, Balakasi K, Phiri K, Chikuse E, et al. Core components of male-specific person-centred HIV care: a qualitative analysis from client and healthcare worker perspectives in Malawi. BMJ Public Health. 2024;2:e001100. 10.1136/bmjph-2024-001100. 40018627 10.1136/bmjph-2024-001100 PMC11816952

[34] Zezai D, Van Rensburg AJ, Babatunde GB, Kathree T, Cornick R, Levitt N, et al. Barriers and facilitators for strengthening primary health systems for person-centred multimorbid care in low-income and middle-income countries: a scoping review. BMJ Open. 2024;14:e087451. 10.1136/BMJOPEN-2024-087451. 39608990 10.1136/bmjopen-2024-087451 PMC11603689

[35] Kredo T, Cooper S, Abrams AL, Muller J, Schmidt BM, Volmink J, et al. Building on shaky ground’—challenges to and solutions for primary care guideline implementation in four provinces in South Africa: a qualitative study. BMJ Open. 2020;10:e031468. 10.1136/BMJOPEN-2019-031468. 32474422 10.1136/bmjopen-2019-031468 PMC7264636

[36] Jensen N, Lund C, Abrahams Z. Exploring effort–reward imbalance and professional quality of life among health workers in Cape Town, South Africa: a mixed-methods study. Glob Health Res Policy. 2022;7:1–16. 10.1186/S41256-022-00242-6/METRICS. 35227327 10.1186/s41256-022-00242-6 PMC8885139

[37] Ahmed S, Chase LE, Wagnild J, Akhter N, Sturridge S, Clarke A, et al. Community health workers and health equity in low- and middle-income countries: systematic review and recommendations for policy and practice. Int J Equity Health. 2022;21:49. 10.1186/s12939-021-01615-y. 35410258 10.1186/s12939-021-01615-y PMC8996551

[38] Okello DRO, Gilson L. Exploring the influence of trust relationships on motivation in the health sector: a systematic review. Hum Resour Health. 2015;13:16. 10.1186/s12960-015-0007-5. 25889952 10.1186/s12960-015-0007-5 PMC4384237

[39] Aberese-Ako M, Van Dijk H, Gerrits T, Arhinful DK, Agyepong IA. Your health our concern, our health whose concern?’: Perceptions of injustice in organizational relationships and processes and frontline health worker motivation in Ghana. Health Policy Plan. 2014;29:ii15–28. 10.1093/heapol/czu068. 25274637 10.1093/heapol/czu068 PMC4202923

[40] Beres LK, Underwood A, Le Tourneau N, Kemp CG, Kore G, Yaeger L, et al. Person-centred interventions to improve patient–provider relationships for HIV services in low-and middle-income countries: a systematic review. J Int AIDS Soc. 2024;27:26258. 10.1002/jia2.26258/full. 10.1002/jia2.26258 PMC11090778 38740547

[41] South Africa launches 1.1 million HIV campaign to close treatment gap [Internet]. Brazzaville: World Health Organization; 2025 Feb 25 [cited 2026 Apr 8]. https://www.afro.who.int/countries/south-africa/news/south-africa-launches-11-million-hiv-campaign-close-treatment-gap-0.

[42] Grimsrud A, Wilkinson L, Raphael Y, Tshabalala S, Moses AK, Hassan F, et al. Securing our HIV response: The PEPFAR crisis in South Africa. South Afr Med J. 2025;115:e3216–3216. 10.7196/SAMJ.2025.V115I3.3216. 10.7196/SAMJ.2025.v115i3.3216 41378515

