# Supplementary material for: “It’s not easy, but we need to support them so they don’t get tired on the way”: provider perspectives on delivering HIV services for people returning to HIV care in Cape Town, South Africa
Source: BMC Health Serv Res. 2026 Jun 9;26:1126. doi: 10.1186/s12913-026-14898-0 (PMC13474612; doi:10.1186/s12913-026-14898-0)
Supplement: Supplementary file 1 [file 12913_2026_14898_MOESM1_ESM.pdf]

In-depth interviews with key stakeholders: Interview Guide

INTRODUCTION

Thank you for agreeing to this interview. As a reminder, we will not record your name in this interview but will use a unique study number to identify you. We will ask for your role/position to help us understand the results. If you mention any names as we talk, we will make sure that the name is removed from the transcript of this interview. This is not an assessment of your work in any way and what you tell us will not be shared directly with anyone. We hope to really understand the services for patients who return to HIV care after an interruption and any challenges you experience.

SECTION A: Demographics

Date of interview	
Time of interview	
Interviewer name	
Participant ID	
Facility	
Any notes on interview setting	
Demographic information	
What is your age?	_____________years
What is your gender?	Female<br>Male<br>Other
What is your job title?	____
What organisation do you work for?	____
How long have you worked in this position?	__________years _____________months
How long have you worked in HIV care and treatment services (overall)?	__________years _____________months
List any qualifications and training you have related to you work (degrees, certificates, diplomas)	____<br>____<br>____<br>____<br>____<br>____

SECTION B: Welcome Back Campaign

1. Please tell me what you understand by "the welcome back campaign".
    a. Describe any activities related to this campaign that you're aware
    b. How are involved with this campaign?
2. From your experiences, can you describe what is working well about the Welcome Back campaign?
3. Can you describe what isn't working well or any challenges you've encountered?

SECTION C: Current services for patients who return to HIV care

We're specifically interested in the services provided for patients who interrupt HIV treatment but then return to the clinic. When I talk about patients who return to care, I mean patients who were on ART but stopped for six months or more and are now back in the clinic to re-initiate ART.

4. Can you tell me how your work involves patients who return to HIV care after an interruption?
   [Prompt if needed: this could be providing clinical care or counselling, or in the management of services for patients living with HIV]
5. Can you describe for me how patients who are returning after an interruption are identified in the clinic?
   [Prompt if needed: think about how the facility would know the patient had previously been on ART, who might identify them, etc]
6. Can you describe the typical services that people returning to care after ART interruption get?
    a. Can you tell me which services they would receive on their typical first visit?
       [prompts if needed: what providers would they see, when would they restart ART]
    b. Can you tell me what the usual follow-up schedule would be in the first 6 months including clinical care, ART dispensing, counselling and support, and viral load testing?
7. Please can you describe the typical counselling provided for patients who return to restart ART after an interruption.
   [prompts if needed: how many counselling sessions, who provides the counselling and where does this happen, what topics are covered]
8. Please can you describe where returning patients usually receive their HIV care in the first 6 months after returning.
    a. Can you tell me when and how they become eligible to join a club or other differentiated model of care?

9. Please describe the ART adherence and retention support a patient might receive after they return to HIV care.
10. Can you tell me how the care for a patient returning after an ART interruption differs from the services for patients who are newly starting ART for the first time?

SECTION D: Experiences

11. Could you tell me, in your opinion:
    a. What are the key reasons patients drop out of HIV care?
    b. What are the key issues that prevent patients returning to the clinic after being out of care?
    c. What are the key issues that prevent patients staying in care after they return to the clinic?
12. Can you describe your feelings towards patients who interrupt their treatment and return to the clinic?
13. In your experience, can you tell me how patients who have previously interrupted care do with their ART adherence and retention after they return?
14. We are interested in your experience with the services for patients who interrupt their treatment and return to the clinic.

    Thinking about providers and care for returning patients:

    a. Please describe any ways you have experienced services for returning patients working well for providers.
    b. Please describe any challenges you are aware of for providers.
    c. Can you tell me any ways you think the services for returning patients could change to make things easier for providers?
       [Prompt: What could be done differently? What could be strengthened?]

    Thinking about the patients and care for returning patients:

    d. Please describe any ways you have experienced the services working well for patients.
    e. Please describe any challenges you are aware of for patients.
    f. Can you tell me any ways you think the services for returning patients could change to make things easier for patients?
       [Prompt: What could be done differently? What could be strengthened?]
15. In your opinion, how should adherence and retention support be for patients who return to HIV care after an interruption.

SECTION E: Flexibility

16. In research among patients who have interrupted HIV treatment one key issue that is raised is the need for flexibility from HIV services, often due to mobility/travel, working hours or other life circumstances. Flexibility could cover a range of things such as where services are obtained, dispensing duration, clinic hours, etc.
    d. Can you describe any ways in the which your clinic/service is offering flexibility to patients?
    e. Can you describe any ideas you have about how HIV services could be more flexible to the needs of patients in the future?

SECTION F: Closing

17. Is there anything else you would like to share with us about services for patients who return to HIV care after a treatment interruption?
18. Please can you give me the details of anyone else you think we should talk to about this topic?

Thank you so much for talking to us today. Your experiences and views are exactly what we are interested in.

END OF INTERVIEW SCHEDULE
