# Supplementary material for: “It’s not easy, but we need to support them so they don’t get tired on the way”: provider perspectives on delivering HIV services for people returning to HIV care in Cape Town, South Africa
Source: BMC Health Serv Res. 2026 Jun 9;26:1126. doi: 10.1186/s12913-026-14898-0 (PMC13474612; doi:10.1186/s12913-026-14898-0)
Supplement: Supplementary file 3 [file 12913_2026_14898_MOESM2_ESM.docx]

Supplemental Table 1. Extended exemplar quotes organised by theme as presented in the manuscript text.

| Themes | Key codes | Extended quotes |
| --- | --- | --- |
| Structural: Work infrastructure | Staff shortage  Provider roles  Training needs  Workflow | … absenteeism of staff. Let me give an example: We’ve got booked patients that are 110 for today. Then the turnout of the staff… I’ve got only 2 sisters. That will mean, if I’ve got an extra load, it was… A sister can only see up to 35. How to do it now… The 70 out of the 110 that we have got… There’s an extra… extra patients that I don’t know, who will see them. They must stretch themselves. And that thing doesn’t make them very nice…They’re not happy at all at that time. And if they overstretch themselves then, be sure tomorrow they’re not coming back. Because that is burnout for them. So that is a problem. And you think on the other hand you’ve got patients that are unbooked. They want to be seen. They need to be seen. And when you say to them: We are not able to see you today… You are [seen as] those bad people… [as though] I don’t want to help them… Like today we’ve got only 1 doctor who’s here. The doctor said: I will only see the appointments. The non-appointments I cannot see. Patients are not impressed. (Nurse Manager, clinic 1)  They say you should do the counselling in 10 minutes or 15 minutes but when you sit with the client that is not the case… because you’re not just doing the HIV counselling, you’re doing more than that you understand? Because someone will tell you why they defaulted and their challenges or their problems at home… Sometimes the doctors or the nurses will say, “no, you’re spending too much time with the patients” you see… And now for us a counsellor, it’s a need; we see that for us, it helps the patient but for you [the doctor] it’s all about time! (Counsellor, clinic 1)  …And there’s also been a holdup on trainings for the staff… due to COVID related issues. Now we’ve started again. That means now we don’t always have full capacity of staff… We are here but now we must let half of the people go to trainings… because we need training. (Nurse Manager, clinic 1)  Some will tell you that, “No, we wait a long time. It’s too long.” Like coming to the clinic, you have to spend the whole day in the clinic and then after that you still have to go to the pharmacy, after waiting long here. So, that is what they will tell you… (Nurse, Clinic 1)  [The Welcome Back nurse] is not always available, they [patients] will be seen by someone else and that's another disappointment, they will see that they are not welcomed. I try to assist when they are not around but it's not going to work because I am working through an agent. I might be here this month and not be here again next month. (Nurse, clinic 2)  They [the clients] don't know the system, they don't know that they will be fast tracked, some lie and don't say they have interrupted treatment. They first go and test and bring a new letter. You only realize this patient was already on treatment when you capture it. Those are the challenges because they are scared of how they will be treated after an interruption… Others would say I was scared because you shout, so I decided to be a new patient. (Administrative Clerk, clinic 1)  It seems like males don't want to lose their work, they see work as the most important thing and default on their treatment. They only come to the clinic when they see things are getting worse or start developing TB. Then it looks like we aren't caring for them because when they come back with TB symptoms, we can't just start them on ARVs. You have to screen them and do all those things; they disappear again and it looks like we don't care. (Nurse, clinic 2) |
| Structural: Physical infrastructure  Structural: Information Technology (IT) infrastructure | Lack of computers and internet access  Limited physical space  Patient folder filing and coordination  Linked information systems | A lack of computers… we have to retrieve the results from our phones… because we’ve got too few PC’s… we end up losing our [personal phone] data instead because… to save time. Because those results, we need in order to see the direction [for the patient]. (Nurse, clinic 1)  Let me talk about space, there is no space…The people in that room, we are six. Maybe one has come to me and has been recruited at the clinic and another one had come because they defaulted, another one for restarting… So, we are all using the same space… How do you counsel a person in that room when there are six of us? (Counsellor, clinic 1)  Duplicated folders is the main issue [when the primary folder is not found]… And each and every one [staff] is pushing around this folder of a duplicate… Not interested to see it because there are lots of things that aren’t there. Information that is missing. (Nurse Manager, clinic 1)  …the only thing is that if a client comes and there's nobody who will be able to locate their folder. Because now you can't see a client without a folder because they need all that information. Especially a client who has been on treatment before, you want to have the previous information. (Nurse, clinic 2)  Number one [problem] for me is the staff. Secondly, I think it's the programs, the system [information systems] that we are using don't link into each other. For example, if you took treatment at [another clinic] I can't see, I don't have a link. I can't see that you were talking about treatment there. So, I think there must be a link to the two. Actually nationally, because if the systems are linking nationally at least when a patient comes from the [another province] and doesn't have a transfer letter at least I know that when I open the system I will see where they are from. (Administrative clerk, clinic 1) |
| Culture: Human equality  Culture: Recipient-centeredness | Provider intention Provider response | I applaud those who return because this journey is tough. I can imagine taking treatment very often. They need to be loved, they need to be cared for, they need to be told that we care for you. You are our community, you are our sisters, brothers and mothers and our children. So, they really need that treatment to show that we love them and support them. It's not easy, but we need to support them so they don't get tired on the way. (Administrative clerk, clinic 2)  What I will say is, there are times that I feel [Sigh] but no man… why like a person could default like this then? But even then, even in my absence I will never… they can never say I get angry, but inside then I feel: but oh no, why like this? Like today I had someone like that. But I said, “Sisi just tell me what is the problem?” Because when you feel that… this is an adult. Why could a person, every time, become pregnant, stop treatment, restart… like for the third time, you know? But then… then again, I think there are a lot of things going through people. (Nurse, clinic 1)  …the staff attitude I would say it still persists somewhere somehow even though some people will say we are not doing that; we are not like that... We still get one or two who are still shouting at the clients and I think none of us would want to be shouted at whether [or not] I have done something wrong. I want somebody to speak to me in a nice way… So, we need to have a listening ear to clients for the problems or challenges that they might have encountered and try to engage with them so that we shouldn't have the same problem and go back to defaulting their treatment… (Nurse, clinic 2)  … They know that they will be shouted at at the clinic if they have defaulted on their medication. I think it takes them lots and lots of courage to come back… From the morning, the patients are shouting because they have been here too long, and we are also worked up and then someone else comes and they need counselling and I am still in that mood from being shouted at… Or the patients are rude and everything, you understand? So, sometimes some of us are not able to correct themselves and understand that this is work, it is not the same person that was shouting at you. This is someone else who needs the attention you give. (Counsellor clinic 1) |
| Relational connections  Communication | Collaboration  Leadership support  Value  Inclusion of implementing partners | They [patients] come with different reasons… As counsellors we find out what the problem is and give them advice… We say we are a team of five in their treatment journey… It's the client, treatment buddy, doctor, counsellor and a nurse. So, they need to know that in order for their lives to be better, we should be a team of five. They also need to be involved in order for their lives to be good. (Counsellor, clinic 2)  We are working on patients and they’ve got the temporary folders at the reception… and the reception would just leave the folders at 3 o’clock and then they vanish. Then we sit down with this new management… This is done with the reception [with the administrative clerks] … and come up with ideas. How about we do this? We try this and see if this can work. Because if it’s work for you then it will work for me. So, as a team it will work for all of us. Therefore, I think that is the new strategies that we are using… because you see, now we are 5 o’clock and no patients. Because of those strategies… I think that they are working. (Nurse, clinic 1)  They [sub-district leadership] always emphasize, they always report that: You’ve got a high number of defaulters. What is happening? What interventions must we apply? Because it’s not like they’re coming up with plans… like, what can we do now? Instead, they carry on reporting: Why are you having a high number of defaulters? What is happening? Are you still having the MDTs? But we are actually having these things… We’ve got them in place. There’s no proper intervention from them, like, “What can we do to assist in order to decrease the number of defaulters?” Instead, they want us to come up, again, with a plan. What are we going to do, again? But we… it’s not like we’re folding our arms. We are actually doing something. But it’s not yielding results - what we are doing. (Nurse manager, clinic 1)  … they have staff meetings that we are not invited to… it’s almost like when they come to us as counsellors, it’s always complaints more than acknowledgement… And let’s say it is the next financial year, that’s when they want to include something that seems like it will accommodate patients because it comes from the organization, not counsellors, whereas as counsellors, we want to be part of that. (Counsellor, clinic 1)  At the moment we are allowing all patients that have defaulted or that have interrupted treatment, to come back and also access treatment. And we make it easier, also, for them because we’ve got a sister [nurse] that is specializing on welcoming them back. But the main issue, because of the COVID-related issues, is the access at the gate. Sometimes they’ve been told at the gate… because another patient would say, “I’m sick, I came here 2 days ago, then I was told I don’t have an appointment, so I couldn’t access [the clinic].” …we don’t see people by dates, but now they will turn you [away] at the gate. (Nurse manager, clinic 1) |
| Inter-facility transfers | Treatment history  Transfer letter  Geographic mobility  Experienced and anticipated stigma | As I said our patients are very mobile, they will move from one area to another. They relocate and then leave and not come back to request a transfer letter… because when you get to the other side, wherever you have moved to, if you don't produce a transfer letter it's difficult for them to say what treatment are you on and whether to continue or to restart you on treatment because they don't know exactly what treatment you are using. (Nurse, clinic 2)  As I said our patients are very mobile, they will move from one area to another. They relocate and then leave and not come back to request a transfer letter… because when you get to the other side, wherever you have moved to, if you don't produce a transfer letter it's difficult for them to say what treatment are you on and whether to continue or to restart you on treatment because they don't know exactly what treatment you are using. (Nurse, clinic 2)  People move places, maybe someone has moved to another province without a transfer letter and they were not accepted there without it, maybe they didn't even bring the clinic card or any proof that they are on treatment… So, the movement is also a problem… So, it depends on the clerk assisting the patient because someone would say it's not my problem that they don't have a letter. (Administrative Clerk, Clinic 1)  They come here and give us local addresses whereas they don't live here in the area because they like this clinic. Maybe they live in [another area] and we can't go there. So, we ask them to tell us the place they wake up every day so that we know where to find them when they don't come to the clinic. If they live in [another area] they have to tell us their nearest clinic so we contact the clinic to recall them for us when we need them. (Administrative clerk, clinic 2)  … And then there’s someone at reception that heard that they got shouted at and they want documents and you do have your documents like ID but then now you don’t have an address, maybe you live in a shanty town “hayi (exclaiming) we also need it [an address], we are not going to be able to.” You see that?... Or maybe they prefer that “I used to take my treatment from a different facility and now I want to take it from this facility.” Right, they get there and they [the clerk] can see on the system… Maybe I want to get my treatment now the system is saying “go back and get a transfer!” How will they come back? (Counsellor, clinic 1) |
